# A survey of *Angiostrongylus* species in definitive hosts in Queensland

**DOI:** 10.1016/j.ijppaw.2015.06.003

**Published:** 2015-07-14

**Authors:** Mahdis Aghazadeh, Simon A. Reid, Kieran V. Aland, Angela Cadavid Restrepo, Rebecca J. Traub, James S. McCarthy, Malcolm K. Jones

**Affiliations:** a School of Veterinary Science, University of Queensland, Gatton, QLD 4343, Australia; b QIMR Berghofer Medical Research Institute, Brisbane, QLD 4006, Australia; c School of Public Health, University of Queensland, Herston, QLD 4006, Australia; d Queensland Museum and Sciencentre, QLD 4101, Australia; e School of Population Health, Australian National University, ACT 0200, Australia; f Faculty of Veterinary and Agricultural Sciences, The University of Melbourne, Parkville, VIC 3052, Australia

**Keywords:** *Angiostrongylus cantonensis*, *Angiostrongylusmackerrasae*, Rat lungworm, Australia, *Rattus rattus*, Southeast Queensland

## Abstract

Despite the recent sporadic reports of angiostrongyliasis in humans, dogs and wildlife in eastern Australia there has been no systematic study to explore the epidemiology of *Angiostrongylus* spp. in definitive and intermediate hosts in the region. Little is known about the epidemiology of *Angiostrongylus* species in the definitive host in southeast Queensland, since the only survey conducted in this region was performed in the late 1960s. In this study, free-living populations of *Rattus* spp. were sampled and examined for the presence of adult and larval *Angiostrongylus* in the lungs, and of larvae in faeces. The prevalence of infection with *Angiostrongylus* spp. was 16.5% in *Rattus* spp. trapped in urban Brisbane and surrounds. This prevalence is much higher than estimates of earlier studies. This highlights the possible risk of zoonotic infection in children, dogs and wildlife in this region and indicates the necessity for public awareness as well as more detailed epidemiological studies on this parasite in eastern Australia.

## Highlights

• The prevalence of *Angiostrongylus cantonensis* in urban populations of feral rats in Southeast Queensland was 16.5%.
• A seasonal pattern was observed for the occurrence of *Angiostrongylus* infection in *Rattus* spp.
• *Angiostrongylus* spp. was not found in *Rattus* spp. of North Queensland.

## Graphical abstract

**Figure:**
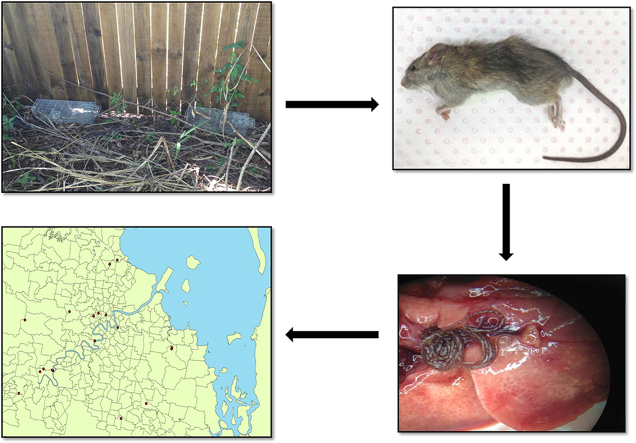

## 1 Introduction

*Angiostrongylus* is a genus of nematode belonging to the superfamily Metastrongyloidea. The genus contains species characterised by a two-host life cycle that always involves a terrestrial and aquatic mollusc as intermediate host (Carreno and Nadler, 2003). All species of *Angiostrongylus* live in the arteries of their definitive host and some have tropism to the central nervous system (CNS) in at least at one stage of their lifecycle in the mammal host. The well-studied lungworm, *Angiostrongylus cantonensis* causes severe and sometimes fatal neurologic disease in accidental hosts, including humans, domestic animals and wildlife. In humans, the clinical features involving CNS, include severe headache, radiculomyopathy and paralysis of cranial nerves (Cooke-Yarborough et al., 1999), while in dogs, clinical signs include hyperesthesia, hind limb paresis and death (Mason et al., 1976). Definitive hosts are infected by the ingestion of infected molluscs containing third-stage larvae of the parasite (Bhaibulaya, 1991) or the mucus secreted from infected mollusc (Heyneman and Lim, 1967). For accidental hosts, it was thought that ingested *Angiostrongylus* larvae migrate only as far as the brain. However, a recent study showed that larvae may also continue to migrate to the pulmonary circulation and complete their development into adults in human (Lindo et al., 2004; Cui et al., 2011).

Another species of rat lungworm, *Angiostrongylus mackerrasae* also occurs in Australia. Although there is no direct evidence that *A. mackerrasae* infects humans, it has recently been reported as a cause of severe lung pathology in a native flying fox (Pteropus alecto) (Mackie et al., 2013). So far definitive speciation of parasites that have caused human disease has been attributed to *A. cantonensis*; however, this assumption needs to be confirmed due to occurrence of *A. mackerrasae* in Australia.

Although terrestrial molluscs are not commonly eaten as food in Australia, isolated incidences of individual cases of human neuro-angiostrongyliasis have occurred due to the accidental or voluntary (as a bet) ingestion of an infected mollusc (Senanayake et al., 2003; Blair et al., 2013; Morton et al., 2013). In particular, *A. cantonensis* has been recognised as a cause of significant disease, especially in children (Li et al., 2001). In the last few years, human cases have been reported from New South Wales (Senanayake et al., 2003; Spratt, 2005; Blair et al., 2013; Morton et al., 2013), raising concerns that neuro-angiostrongyliasis may be emerging as a more significant public health issue in eastern Australia.

Current knowledge of the epidemiology *Angiostrongylus* spp. throughout Australia is limited to small surveys of rats conducted in Queensland in the 1950s and 1960s (Mackerras and Sandars, 1955; Bhaibulaya, 1968), a survey of rodents in the Gulf of Carpentaria in late 60s (Dunsmore, 1968), two surveys of dogs in Queensland and New South Wales (Mason, 1987; Lunn et al., 2012) and a survey of rats in Jervis Bay in New South Wales between 2003 and 2005 (Stokes et al., 2007). Most of the above rodent surveys were performed in non-urban areas, distant from human habitation and, therefore, may not reflect the potential risk of human infection.

The prevalence, distribution and the potential hotspots for transmission of *Angiostrongylus* spp. are unknown in eastern Australia. Hence this study aimed to place a step forward to investigate the presence of *Angiostrongylus* infection in the definitive host among randomly selected populations of *Rattus* spp. in southeast Queensland where the parasite has been previously reported.

## 2 Materials and methods

### 2.1 Sample collection

**Fig. 1:**
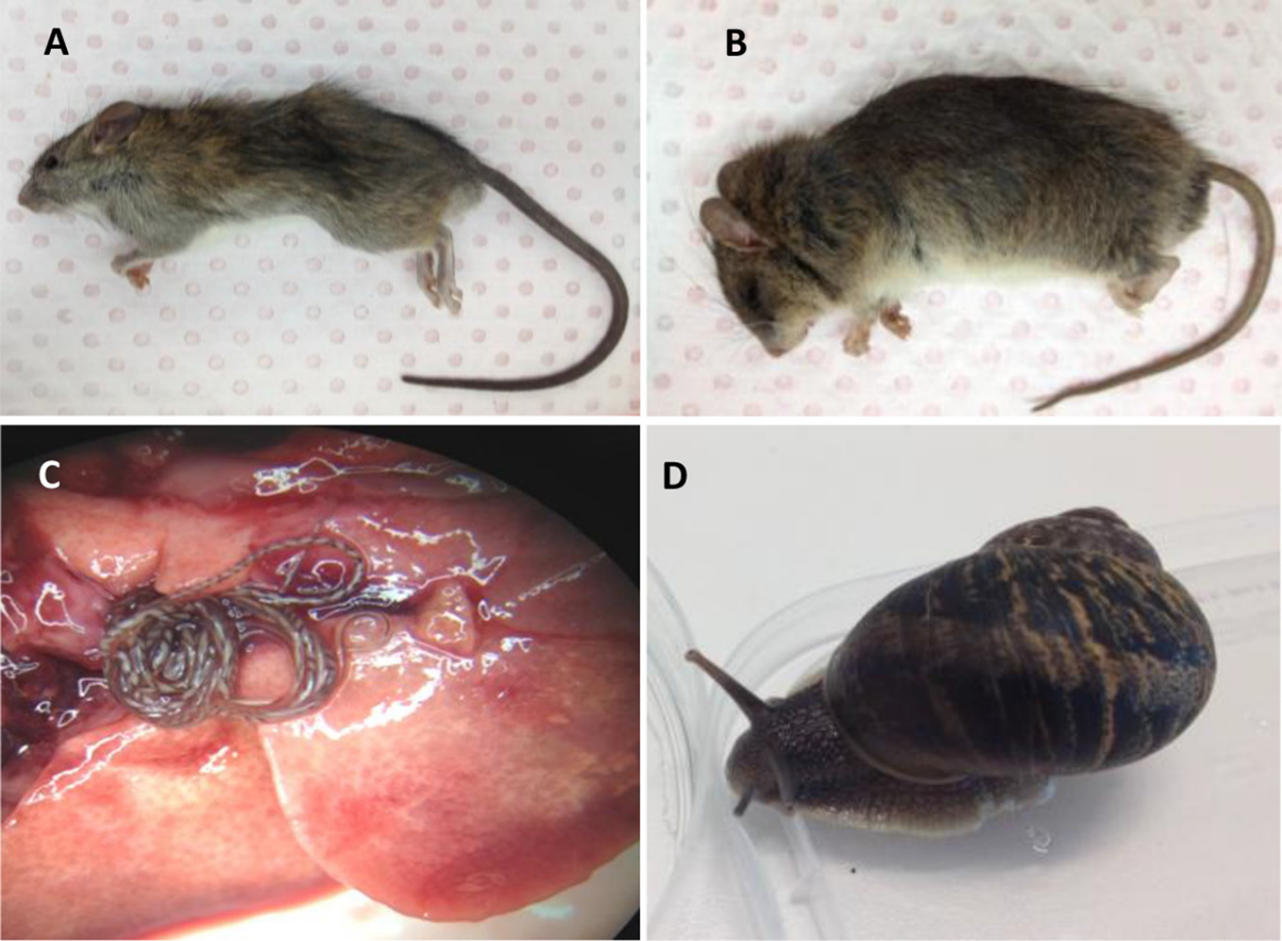
Images of two principal *Rattus* spp., *Angiostrongylus cantonensis* and its putative intermediate host from a survey conducted in Brisbane, Australia, 2012–2014. A: *Rattus rattus* was the most prevalent species of rat found in this survey. B: *Rattus fuscipes*. C: Adult *A. cantonensis* in pulmonary arteries of *Rattus rattus*. D: *Helix aspersa* found is Brisbane harbouring *Angiostrongylus* larvae.

This study was approved by the QIMR Berghofer animal ethics committee (Project Ethic No: A1208-607M). Introduced rats (*Rattus rattus* and *Rattus norvegicus*) and native rats (*Rattus fuscipes* and *Rattus lutreolus*) were trapped in different areas in and around Brisbane (mostly urban areas) between June 2012 and January 2015 (Fig. 1) (under permit from the Department of Environment and Heritage Protection of the Queensland Government: WIS12109412. Brisbane is a city located in southeast Queensland, Australia where the average annual temperature ranges between 16.2 °C and 26.4 °C and the average annual rainfall is approximately 1149.1 mm. An additional survey was conducted in Cairns, north Queensland in May 2014 where the annual temperature ranges between 20.8 °C and 29.0 °C and the average annual rainfall is approximately 2010.7 mm. Trap sites were chosen non-randomly based on the reported presence of *Rattus* species.

**Fig. 2:**
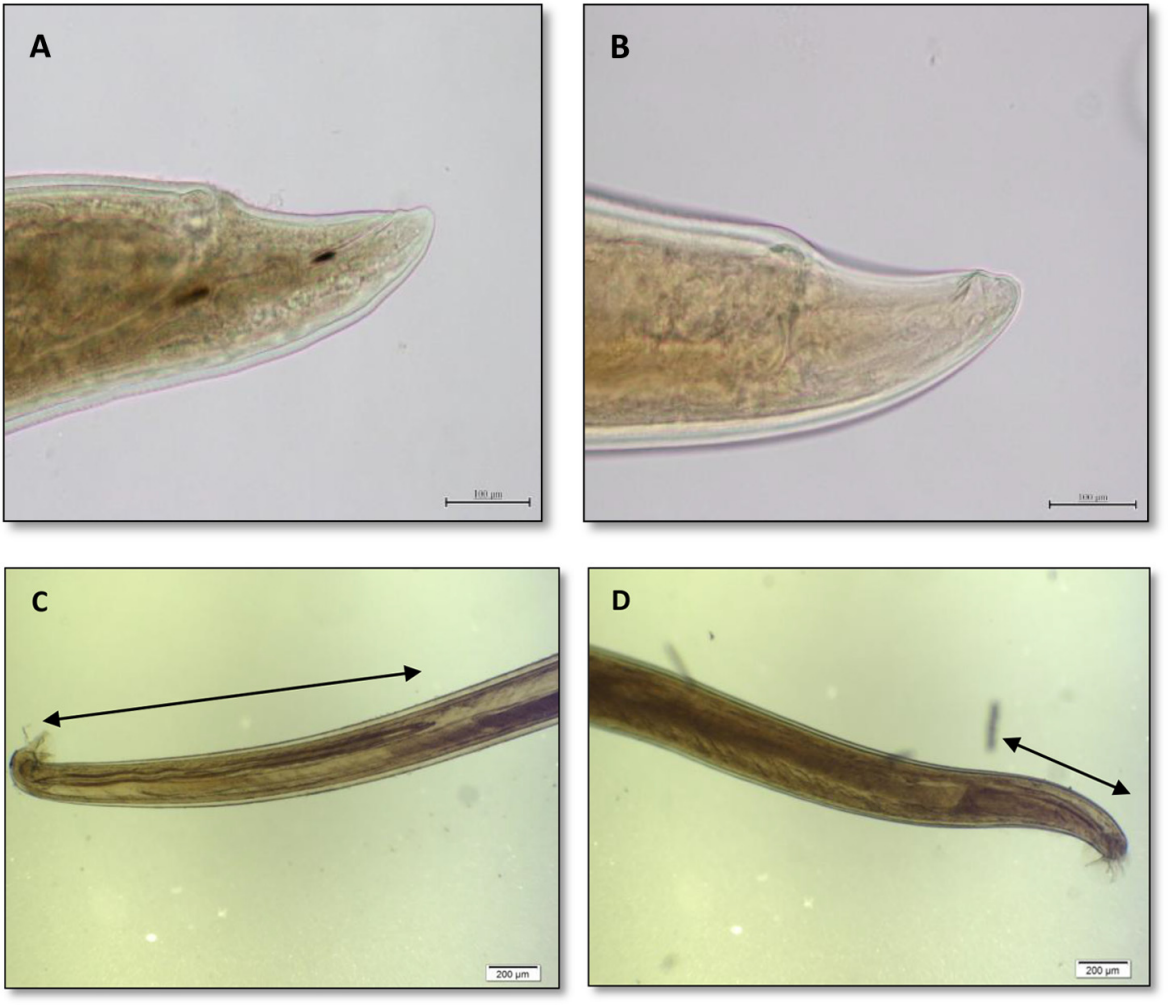
Adult *Angiostrongylus* spp. recovered from pulmonary arteries of *Rattus rattus* (*A. cantonensis*) and *Rattus fuscipes* (*A. mackerrasae*) trapped in Brisbane, Australia, 2012–2015. A: Posterior end of female *A. cantonensis*; B: Posterior end of female *A. mackerrasae*: The distance between vulva and posterior end is very similar between the two species; C: Male *A. cantonensis* and D: Male *A. mackerrasae*: Spicule length is about 2.5 times longer in *A. cantonensis*.

Wire cage traps baited with peanut butter and rolled oats were placed along walls or fences at least 2 m apart for at least one week with nightly rebaiting. The location of the traps was recorded using spatial coordinates obtained by global positioning system (GPS). Each trapped rat was euthanized on site using a portable carbon dioxide chamber. Rats were then transported to the laboratory where the lungs and pulmonary vasculature were examined for the presence of adult lungworms. Each adult male *Angiostrongylus* recovered from lungs of positive rats was examined to determine the species based on the spicule length of male worm described by Bhaibulaya (1968) as illustrated by Aghazadeh et al. (2015) and here (Fig. 2). All the recovered *Angiostrongylus* spp were preserved in 70% ethanol for later molecular testing. Samples of kidney, liver, spleen and faeces were also collected from each rat and stored at −20 °C for further analyses. The dimensions of the spleen were recorded for each rat and splenic size was calculated using the formula described by Araujo et al. (2014) in order to investigate any association between *Angiostrongylus* infection and splenomegaly.

The prevalence of *Angiostrongylus* spp. in intermediate hosts was determined by sampling mollusc species from different areas in Brisbane and surrounds, and digested using an artificial gastric solution (2 g of 1:3000 Pepsin: Amresco LLC, OH, USA and 8 ml of 36% HCl in 1 L of water). The digests were then visually examined using light microscopy to identify first to third-stage larvae of *Angiostrongylus* spp.

### 2.2 Data analysis

The associations between *Angiostrongylus* infection, the worm burdens and rat body weight, maturity for both rodent groups (introduced or native) were evaluated using the Chi-square test (for dichotomous variables) and Independent T-test (continuous variables) using IBM SPSS Statistics for Windows (Version 22.0. Armonk, NY: IBM Corp.) with a 95% confidence level. Maturity of rodents was determined by using 100 g as the threshold between adult (>100 g) and juveniles. Two individual *R. fuscipes* were excluded from the analysis because they were euthanized following microscopic examination of their faeces to confirm infection.

An administrative boundary map of Queensland was downloaded from the DIVA-website (DIVA-GIS, 2012) and linked by location to the geo-referenced angiostrongyliasis dataset using the Quantum GIS software package, version 1.8.0, ‘Lisboa’ (Quantum GIS Development Team, 2012). The mean prevalence of infection and binomial 95% confidence intervals were calculated. Statistical significance of any observed differences in proportion were assessed using Chi-square test at a 95% confidence level.

In addition, the calculated size of spleens were compared between the infected and non-infected rats using Mann–Whitney test (Graph Pad Prism, version 6) in order to investigate an association between spleen size and *Angiostrongylus* spp. infection.

## 3 Results

A total of 402 rats were trapped in Brisbane and its surrounds (375) and in North Queensland (27). The majority of trapped rats were introduced, belonging to *R. rattus* (325/340: 95.5%) with smaller numbers of *R. norvegicus* (15/340: 4.5%). Of the 62 native rats that were trapped, the majority were *R. fuscipes* (55/62: 88%) followed by *R. lutreolus* (5/62: 8%) and *Rattus sordidus* (2/62: 3%) (Table 1).

A significantly higher proportion of adult introduced rats were found to be infected with *Angiostrongylus* spp. compared to juvenile introduced rats (p = 0.012) (Table 2). A higher (not significant) proportion of adult native rats were infected with *Angiostrongylus* spp. compared to juvenile native rats. The prevalence of *Angiostrongylus* spp. infection was higher (not significant) in native rats (26.7%; 95% CI 16.1–39.7%) compared to introduced rats (16.5%; 95% CI 12.7–20.8%) (p = 0.68).

A total of 47 and eight adult male worms were recovered from 29 introduced and five native rats, respectively. An overlap was observed in the differences between adult female worms (length of vulva, distance from vulva to the posterior end and the distance from anus to the posterior end), similar to the range described by Bhaibulaya (1968). Hence, the species identification was not possible based on only female worms. Mixed infections were not observed in this study.

The mean bodyweight of all infected rats was significantly higher compared to uninfected rats (p = 0.44) (Table 2). All infected juvenile rats were significantly heavier compared to uninfected juvenile rats (p = 0.001). The mean bodyweight of infected juvenile introduced rats was significantly higher compared to uninfected introduced juvenile rats (p = 0.007). There was no significant difference in the body weight of infected and uninfected adult introduced rats. Infected adult native rats had a lower (not significant) bodyweight compared to uninfected adult native rats (p = 0.085). Also, there was no difference in the body weights of infected and uninfected adult introduced rats and juvenile native rats. The mean worm burden was higher (not significantly) in introduced rats compared to native rats (p = 0.282) and in all adult compared to all juvenile rats (p = 0.007). Among introduced rats species, adults had more worms than juveniles (p = 0.024) but no age differences in intensity were seen among native rat species.

**Map 1:**
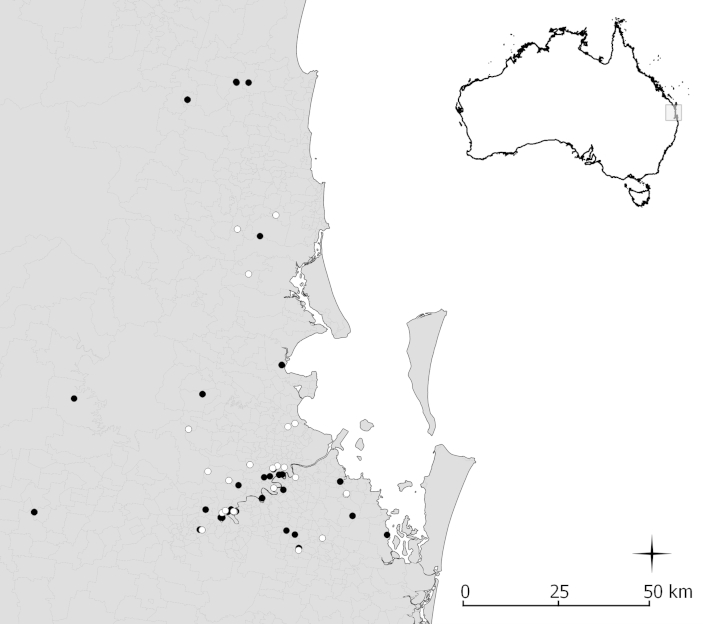
Areas in Queensland, Australia (S23.0, E143.0) that sampling was performed for a survey of introduced and native *Rattus* spp for infection with *Angiostrongylus* spp., 2012–2015. NB: white circles represent locations where infected rats were trapped and black circles where uninfected rats were trapped.

Most of the infected rats were trapped in areas that were in close proximity to either a creek (stream) or to the Brisbane River (Map 1 and Table 3). A seasonal pattern was also seen for the presence of infection in introduced rats where a higher percentage of rats were found to be infected among rodents collected between April and July. The *Rattus* spp. collected in North Queensland comprised 5% of the total sample and were all negative for the presence of *Angiostrongylus* spp in the lungs, heart and faeces. Statistical analysis of the spleen showed no significant difference (p = 0.059) in the size of the spleens between the infected and uninfected *Rattus* species.

Ten different species of molluscs were collected from Brisbane and its surrounds (Table 4). Only 1 (*Helix* aspersa) of the 87 individual molluscs collected from the suburb of Moggill was found to be infected with 2 *Angiostrongylus* larvae.

## 4 Discussion

The results of this study revealed a relatively high prevalence of *Angiostrongylus* spp. in rats in urban Brisbane and some surrounding areas, surpassing the two previous surveys of *Angiostrongylus*, conducted in Queensland (Bhaibulaya, 1968) and in New South Wales (Stokes et al., 2007). The high prevalence indicates that a relatively large reservoir and infection pressure exists for dogs and children in Brisbane. Despite the wide distribution of the parasite in urbanized regions of Brisbane city, reported human cases from this part of Australia are rare. However, there is a possibility that the disease is under-diagnosed or if cases are detected, are not reported. One reason for poor diagnosis of angiostrongyliasis is that patients with *Angiostrongylus* infection may not display symptoms characteristic of the disease upon first presentation to clinics. Typical signs, such as blood and CSF eosinophilia, and sero-conversion are not always present in early stages of infection (Tseng et al., 2011). In fact, sero-conversion might never occur in small children (Lindo et al., 2004; Morton et al., 2013). It has also been reported that the clinical signs can be mild and the disease might be self-limiting based on the intensity of infection and the immune response of the patient (Prociv et al., 2000). This suggests that it would be necessary to raise the awareness of clinicians as well as the general public to the presence of this parasite and route of infection. Furthermore, educational programmes such as the school booklet developed by researchers at University of Hawaii (Riddick et al., 2013) may be beneficial in eastern Australia to raise awareness about neural angiostrongyliasis.

The observation that a higher proportion of adult rats was infected and that these rats had a higher intensity of infection with *Angiostrongylus* spp. is in agreement with previous studies on *A. cantonensis* (Chen et al., 2011) and other long lived helminth infections (Woolhouse, 1998; El-Khoby et al., 2000). This most likely reflects that longevity of infection and gradual accumulation of new and repeat infections (trickle infection) in individual animals. It was interesting to note the apparent negative association between bodyweight and intensity of infection only in native adult rats. Whilst this result was not statistically significant it may suggest that native rats are adversely affected by *Angiostrongylus* spp. infection in adulthood.

Furthermore, the small sample size of native rats in this study may have masked a more significant trend in bodyweight. This association was not observed in introduced rats, which are the natural host of *A. cantonensis*. If *A. mackerrasae* is more pathogenic in native rats then it is possible that it has not been present in Australia for the same duration as its native rat host species. However, this disagrees with the hypothesis proposed by Prociv et al. (2000) who suggested that the limited geographic and host range of *A. mackerrasae* (i.e. it is only present in Australia and found only in native *Rattus* spp.) suggests the result of a long co-evolution. This is important because the origin of *Angiostrongylus* spp. in Australia is still unknown and recent studies have shown that *A. cantonensis* and the native *A. mackerrasae* are almost indistinguishable using molecular tools and may represent recently divergent, or diverging species (Aghazadeh et al., 2015; unpublished data). Although hybridization of the two species by Bhaibulaya (1974) suggests that the two species are distinct and can form fertile female offspring.

Southeast Queensland has a subtropical climate with a distinct wet season from November to May and a dry cooler season from June to October. In this study, there is an observed seasonality in the prevalence of *Angiostrongylus* spp. infection, with an observed increase in prevalence one to two months after the start of rainy season in April and May. This may be associated with an increase in the abundance of molluscs during the peak of the wet season in January and February adjusted for the approximate 60 days pre-patent period in infected rats (Bhaibulaya, 1975). Reports of the seasonal occurrence of angiostrongyliasis in humans in New Caledonia (Rosen et al., 1967) and Hawaii (Alicata, 1988) are associated with the peak vegetable-growing season in the cooler months, which was attributed to accidental ingestion of mollusc hidden in vegetables. In these settings, vegetables are imported during the hot, wet season. Lunn et al. (2012) also recognised a seasonality pattern for infection in dogs in New South Wales with a peak in mid autumn to early winter, similar to this study. However, it is difficult to make firm conclusion from the results of this study because sampling was not undertaken uniformly across the whole year. Though, the link between the availability of molluscs for rats to ingest and seasonality seem biologically plausible. Seasonality also can affect the risk of ingestion of the parasite by human and other non-permissive hosts such as dogs.

In conclusion, data obtained from this study confirm the high prevalence of *Angiostrongylus* spp in native and introduced *Rattus* spp in southeast Queensland and indicate the need for raising public awareness about this parasite in this region. The current data could form the basis for the development of a risk map for the presence of *Angiostrongylus* in different suburbs of Brisbane and for further research to investigate risk factors such as the correlation between rainfall and risk of infection with *Angiostrongylus*.

## Funding

Mahdis Aghazadeh is supported by a University of Queensland Postgraduate Award. This research was supported by grants from the ANZ Queensland Community Foundation – Peter and Mary Ellen Stone Memorial Fund, awarded to Malcolm Jones.

## Conflicts of interest

All authors declare that no competing interest exists.

## Figures and Tables

**Table 1:** *Rattus* species examined in this survey and the number of each species harbouring *Angiostrongylus* species.

Rat species	Locality	No. examined	No. Infected	% Positive
*Rattus rattus*	SE and N^a^ QLD	325	52	16
*Rattus norvegicus*	SE and N QLD	15	4	27
*Rattus fuscipes*	SE QLD	53	14	26
*Rattus lutreolus*	SE QLD	5	2	40
*Rattus sordidus*	N QLD	2	0	0

SE QLD = Southeast Queensland; N=North.

a *Rattus* species collected in N QLD did not harbour *Angiostrongylus* species.

**Table 2:** The mean weight and *Angiostrongylus* spp. burden in native and introduced *Rattus* spp. trapped in Queensland, Australia, 2012–2015.

Species	Maturity	Infected			Not infected		Total^a^
		No.	Weight (g)	No. worms	No.	Weight (g)	
*R. fuscipes*	Adult	11	141.1	7.4	26	154.4	37
	Juvenile	3	95.7	2.0	10	88.3	13
*R. lutreolus*	Adult	2	149.2	1.8	2	147.6	4
	Juvenile	–	–	–	1	89.2	1
*R. norvegicus*	Adult	3	176.7	6.5	9	165.2	12
	Juvenile	1	96.6	3.0	3	79.0	4
*R. rattus*	Adult	46	154.9	11.3	195	157.6	241
	Juvenile	6	86.6	5.0	77	74.8	83
*R. sordidus*	Adult	–	–	–	1	150.9	1
	Juvenile	–	–	–	1	61.3	1

a The Weights of 6 native rats were not recorded and therefore was deducted from this table.

**Table 3:** List of areas in Queensland that *Rattus* samples were collected from between 2012 and 2015. Localities with *Angiostrongylus* infected *Rattus* spp are shown.

Suburb	Latitude	Longitude	*Angiostrongylus* spp	Prevalence (%)
Julatten, NQLD	S 16.58631	E 145.33828	–	–
North Cairns	S 16.9036333	E 145.7558924	–	–
Redlynch, NQLD	S 16.955501	E 145.689729	–	–
Atherton, NQLD	S 17.26051	E 145.51797	–	–
Lawes	S 26.406888	E 152.89913	–	–
Cooroy	S 26.40888	E 152.93266	8/23	34.7
Carters ridge	S 26.4551303	E 152.7673416	–	–
Kobble Creek	S 27.25165	E 152.80797	–	–
Nudgee beach	S 27.3154	E 153.1239	–	–
Chermside	S 27.3310247	E 153.0582487	2/6	33.3
Mt Glorious	S 27.34661	E 152.77018	1/30	3.3
The Gap	S 27.442556	E 152.936348	5/10	50
Herston	S 27.44944	E 153.02932	3/5	60
Redhill	S 27.454763	E 152.999868	–	–
Brisbane City	S 27.46888	E 153.0239	–	–
South Brisbane	S 27.46972	E 153.01543	–	–
Auchenflower	S 27.47396	E 152.99036	–	–
Mt Coot-tha	S 27.47653	E 152.97472	–	–
Norman Park	S 27.476723	E 153.059613	1/3	33.3
Ransome	S 27.48811	E 153.18057	–	–
Upper Brookfield	S 27.49804	E 152.90529	4/15	26.6
St Lucia	S 27.50566	E 153.00091	7/38	18.4
Woolloongabba	S 27.510508	E 153.026782	–	–
Capalaba	S 27.52291	E 153.19711	7/13	53.8
Fig Tree Pocket	S 27.532933	E 152.969297	–	–
Bellbowrie	S 27.5637147	E 152.8854058	–	–
Moggill	S 27.569459	E 152.877113	14/84	16.6
Caloundra	S 27.57081	E 152.35273	1/1	100
Sheldon	S 27.5808646	E 153.213923	–	–
Silkstone	S 27.619106	E 152.806867	6/18	33.3
Parkinson	S 27.62073	E 153.03505	–	–
Redland Bay	S 27.6322924	E 153.3074069	–	–
Springwood	S 27.641253	E 153.132301	3/8	37.5
Logan city	S 27.66826	E 153.06856	6/20	30

**Table 4:** List of mollusc species and the areas from which they were collected in Queensland between 2012 and 2013.

Mollusc species	Sampling location
*Bradybaena similaris*	Alderley, Herston, Spring Hill, Birkdale
*Deroceras panormitanum*	Birkdale, Oxley Common
*Hedleyella falconeri*	Mt Tamborine
*Helicarion* sp	Alexandra Hill, Moggill
*Helix aspersa*	Moggill^a^, Herston, Gatton
*Lehmannia nyctelia*	Spring Hill, Moggill, Elimbah
*Limax maximus*	Elimbah
*Stanisicarion virens*	Alexandra Hills, Maleny
*Teriboniophorus graeffei*	Kenmore, Alexandra Hill
*Terrycarlessia turbinata*	Elimbah

a The only suburb in which *Angiostrongylus* spp. larvae was found in molluscs.
